# Exploration of utility of combined optical photothermal infrared and Raman imaging for investigating the chemical composition of microcalcifications in breast cancer

**DOI:** 10.1039/d2ay01197b

**Published:** 2023-02-21

**Authors:** Pascaline Bouzy, Iain D. Lyburn, Sarah E. Pinder, Robert Scott, Jessica Mansfield, Julian Moger, Charlene Greenwood, Ihssane Bouybayoune, Eleanor Cornford, Keith Rogers, Nick Stone

**Affiliations:** a School of Physics and Astronomy, University of Exeter Exeter UK N.Stone@exeter.ac.uk; b Cranfield Forensic Institute, Cranfield University Shrivenham UK; c Gloucestershire Hospitals NHS Foundation Trust UK; d King's College London, Comprehensive Cancer Centre at Guy's Hospital London UK; e School of Chemical and Physical Sciences, Keele University Keele Staffordshire UK

## Abstract

Microcalcifications play an important role in cancer detection. They are evaluated by their radiological and histological characteristics but it is challenging to find a link between their morphology, their composition and the nature of a specific type of breast lesion. Whilst there are some mammographic features that are either typically benign or typically malignant often the appearances are indeterminate. Here, we explore a large range of vibrational spectroscopic and multiphoton imaging techniques in order to gain more information about the composition of the microcalcifications. For the first time, we validated the presence of carbonate ions in the microcalcifications by O-PTIR and Raman spectroscopy at the same time, the same location and the same high resolution (0.5 μm). Furthermore, the use of multiphoton imaging allowed us to create stimulated Raman histology (SRH) images which mimic histological images with all chemical information. In conclusion, we established a protocol for efficiently analysing the microcalcifications by iteratively refining the area of interest.

## Abstract 2

We aim to develop a protocol for efficiently analysing breast microcalcifications and their microenvironment in detail by combining O-PTIR and Raman spectroscopy at the same time, the same location and the same high resolution (0.5 μm).

## Graphical abstract

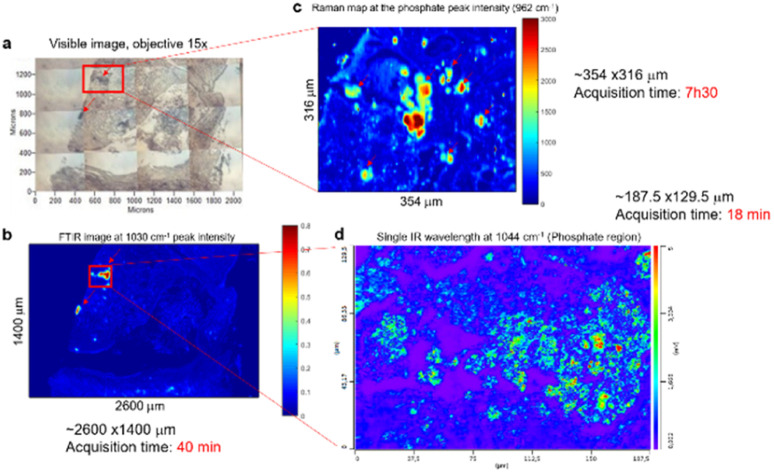

## Introduction

Breast microcalcifications (BMCs) have intrigued radiologists, pathologists and scientists because their formation and maturation will be directly affected by the local tissue physiology, thus they could be an important measure and potential predictor of breast cancer progression. BMCs were described for the first time by Leborgne, as an abnormal mineral deposit in the mammary tissue which could be associated with breast cancer.^1,2^ On mammography, radiologists can detect microcalcifications of sufficient size to be resolved (usually spatial resolution >50 μm per pixel),^3^ although neither their composition nor their surrounding tissue pathology can be ascertained on X-ray. BMCs are classified according to their morphology, their spatial distribution, and their histological location. They are found in a range of pathologies as well as normal breast tissue, although their composition may differ according to the type of lesion: calcium oxalate dihydrate (COD) crystals are mainly found in benign processes whereas hydroxyapatite (Hap) crystals are found in both benign and malignant lesions. Hap crystals appear to be more complex than COD. In fact, Hap crystals could be substituted by different elements at multiple lattice sites in BMCs in different concentration *e.g.*, carbonate ions, sodium ions.^4–7^ These substitutions are possible due to an environment rich in various ions. This could be helpful for diagnosis, as a study by R. Baker *et al.*, suggests that the carbonate level is higher in benign pathologies than invasive cancers^4^ and could be a useful indicator of the nature of the breast pathology. Furthermore, unpublished work by the group and published work by A. Ben Lakhdar *et al.* have demonstrated that this level of carbonate could be heterogenous within a BMC in a ductal carcinoma (DCIS) lesion.^8–10^ Over the past decades, several studies have suggested that the chemical composition of the BMCs is more complex than expected. In fact, under the influence of the microenvironment, other types of phosphate species could be present in the BMCs^5,11,12^*e.g.*, amorphous calcium phosphate,^13^ octacalcium phosphate,^14^ and tricalcium phosphate^15^ and whitlockite.^11,13,16^

However, the formation of BMCs and their possible role in cancer are still not fully understood even if many theories are found in the literature.^17–21^ Furthermore, there is a lack of information about their chemical composition, distribution, morphology, and relationship with surrounding soft tissues.

Scanning electron microscopy (SEM) has highlighted a range of different morphologies but also a heterogeneity within the structure of BMCs.^8,22^ For instance, several studies have shown that some pathological calcifications exhibited concentric ring structures. Gaining chemical information about these structures could help to understand the formation of the pathological calcifications. In this context, it is fundamental to use different microscopic techniques in order to correlate the morphology, the chemical composition and the breast pathology.^22,23^

Vibrational spectroscopy (Fourier Transform Infrared (FTIR) and Raman spectroscopy) has been used for biological and clinical evaluation for several decades, applied to cells, biofluids and tissue sections.^24–27^ It has also been recognised as a powerful tool for identifying BMCs in breast tissues as well as their biochemical composition.^4,13,28–32^

These vibrational spectroscopic tools offer many advantages but also some challenges when applied to breast tissue sections. The traditional FTIR systems have a Mercury Cadmium Telluride (MCT) detector, which covers a large spectral range (∼3900–800 cm^−1^), collecting the information regarding lipids, proteins, and mineral content. Unfortunately, these FTIR systems have a very low spatial resolution, due to the diffraction limit of the technique, 1000 cm^−1^ being equivalent to a wavelength of 10 μm.

There are some alternatives to improve the spatial resolution of IR such attenuated total reflectance (ATR) FTIR spectroscopy. However, this approach needs a close contact between the sample and an ATR crystal, which has potential to cause sample damage. It can result in a resolution improvement, dependent on the refractive index of the crystal, *e.g.* 4 for germanium, reducing the spectral resolution achievable from 5–12 μm to 1.2–3 μm for mid-IR.

In FTIR, the signal-to-noise ratio of the data in the long wavelength region can be impacted by the attenuation from substrates, system optics and the calcification itself, which is highly absorbing compared to biological tissues of the same thickness. Sometimes, the microcalcification size is smaller than the FTIR system can resolve.^33^ In addition, the quality of the data is dependent on the sample preparation *i.e.*, a saturation of the IR signal can be caused by a thickness of only a few microns of highly absorbing phosphate rich mineral.

Raman spectroscopy provides more flexibility in terms of sample preparation and analysis, *i.e.*, liquid or powder samples, or thick tissue samples, as well as an order of magnitude improvement in spatial resolution, with diffraction limited resolution of between 500 and 1000 nm. However, Raman scattering cross-sections are relatively low and intense laser light is required to provide sufficient signals in reasonable timescales. This can lead to the risk that the laser can induce burning due to paraffin residues, ink or the calcifications themselves. This phenomenon is not generally observed with NIR excitation wavelengths in the majority of biological tissues,^34,35^ but appears to be feasible, particularly with 785 nm laser.

In addition to the traditional FTIR and Raman spectroscopy techniques, a new technique has emerged in the literature, which is Photothermal Infrared (PTIR) spectroscopy.^36,37^ This technique is based on utilising the photothermal effect, induced by a tuneable mid-IR laser, and allows the user to achieve as small as nanoscale resolution. This was first demonstrated with the use of atomic force microscopic (AFM) tips being used to measure the thermoelastic expansion of the sample when a tuneable IR laser source matches the absorption frequency/wavelength of the material. Note that while, the lateral resolution of an O-PTIR microscope is equal to 500 nm, the lateral resolution of an AFM-IR microscope is around 50 nm.^38,39^

More recently the technique of optical photothermal infrared (O-PTIR) spectroscopy has been developed, which measures the photothermal response of the sample to pulses of single frequency mid-IR radiation.^40^ The thermoelastic expansion following the resonant absorption leads to a change in the local refractive index of the sample. The photothermal response is detected by a non-destructive visible or NIR laser probe which determines the spatial resolution of the measurement. This results in a submicron spatial resolution (around 500 nm *vs.* 5–12 μm of FTIR)^41,42^ without requiring any contact with the sample, reducing the potentially for sample damage.^43^

The photothermal system offers other advantages; using a series of quantum cascade lasers (QCL), which are significantly brighter sources than a Globar thermal source, to cover the entire fingerprint region (1800–800 cm^−1^). QCLs are also rapidly tuneable to a specific wavelength of interest, enabling relatively fast discrete frequency mapping to be achieved. Vibrational spectroscopy associated with QCL has emerged during the past decades and provides a rapid and highly sensitive approach.^44,45^ The past few years have seen several demonstrations of mid-IR imaging using QCL sources that have been applied in several studies investigating, for example, colorectal disease^45^ and breast cancer.^46^

For the first time a system is available which combines all the advantages of the previous techniques described above with a rapid and sensitive approach and a high resolution. In brief, within our system, the sample is illuminated by a pulsed mid-IR laser. When this IR laser is tuned for a specific wavenumber for a specific IR absorption, a photothermal expansion is created in the sample. These changes are detected a visible or NIR laser probe. Importantly for multi-modal analytical validation, this laser probe enables the simultaneous collection of Raman and IR spectra, at the same location and the same resolution.^41,42,47^

In parallel, a multimodal approach that offer a submicron resolution has been performed. First, the second harmonic generation (SHG) technique has been applied, which is well-known for imaging collagen in biological samples.^48^ Its application to the analysis of BMCs provides new perspectives, as the collagen seems to play a role to discriminate BMCs in different breast pathologies. In fact, as suggest by Shin *et al.*, the level of collagen is higher in fibroadenoma rather than invasive ductal carcinoma, which both have similar carbonate level.^49^ Moreover, two photon fluorescence (TPF), widely used to identify mineral tissues,^50,51^ could help by identifying the BMCs in the breast tissue sections. Finally, stimulated Raman scattering (SRS), a non-linear optical technique, offers the ability to obtain complementary information to Raman spectroscopy as it allows imaging of biological tissues and exploration of their chemical composition with a submicron resolution.^49,51^ Likewise, this multimodal approach generates stimulated Raman histology (SRH) images based on the SRS images for the CH_2_ and CH_3_ bonds (2845 and 2930 cm^−1^, respectively) in good agreement with the H&E images.^52,53^ These images are created based on label-free and non-destructive techniques and could provide faster histological information when standards H&E images are not available.

The objective of this study was to investigate the potential of combined O-PTIR and Raman spectroscopy for analysing breast BMCs, their composition, and the surrounding tissues and to ascertain when this instrument adds additional value over routine vibrational spectroscopic imaging approaches. Furthermore, the complementary nature of stimulated Raman scattering (SRS) and second harmonic generation (SHG) microscopy techniques allow visualisation of the collagen network and the protein distribution in the tissue^50,51^ as well as within the BMC with a very good resolution.^54,55^ By integrating all these techniques, we aim to gain more information about BMCs in different breast pathologies.

## Experimental

### Sample preparation

**Fig. 1:**
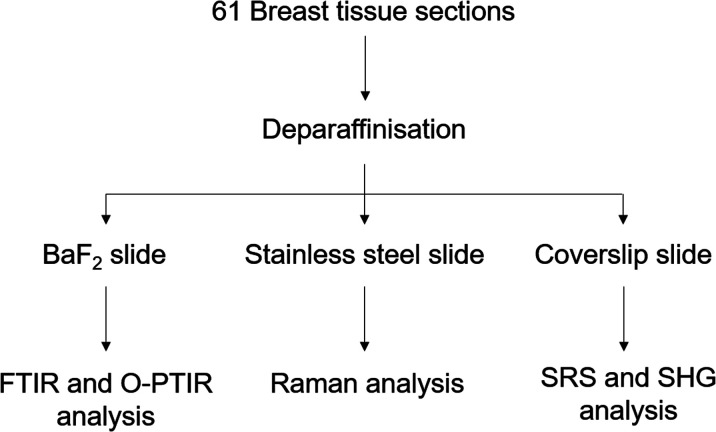
Experimental protocol used to analyse the breast tissue sections using FTIR, O-PTIR and Raman spectroscopy and multiphoton imaging.

Forty-one core biopsy samples and twenty breast surgical excision specimens were analysed. Adjacent sections were cut for specific analyses: one section of 5 μm thickness for a histological analysis with a haematoxylin and eosin (H&E) staining was mounted onto a glass slide, another section of 2 μm thickness was mounted onto barium fluoride (BaF_2_) substrate (Crystran, Poole, UK) for FTIR, Raman and O-PTIR spectroscopy analysis. The last section of 5 or 10 μm thickness was mounted onto a super mirror stainless steel substrate (Renishaw PLC, Gloucester, UK) for Raman and O-PTIR spectroscopy analysis (see Fig. 1).

Prior to the spectroscopic analysis, all samples were deparaffinised using the following protocol: 3 baths of Histoclear (National Diagnostics, USA) for 5 min, following by 15 dips in isopropanol (IPA) 100% (Fisher Scientific, UK), repeated twice, then 15 dips in IPA 90%, 15 dips in IPA 70% and 15 dips in distilled water. The samples were air dried at room temperature before analysis.

### Vibrational spectroscopy

Samples were measured using an Agilent 670 FTIR spectrometer coupled to an Agilent 620 FTIR microscope equipped with a 15× Cassegrain reflective condenser and objective (N.A. 0.62) and a liquid nitrogen cooled MCT-FPA detector (128 × 128 pixels).

Micro-Raman measurements were acquired using a Renishaw InVia confocal Raman microscope, comprising a 50× long working distance objective (N.A. 0.5) for illumination and for collection of the backscattered light, xyz motorized stage, 600 lines per mm grating and CCD camera. The excitation beam was from a diode laser source with 785 nm wavelength. Before each set of measurements, a system calibration was performed using three standards: silicon wafer, polytetrafluoroethylene (PTFE) and green glass.

Simultaneous O-PTIR and Raman measurements were carried out using a mIRage IR microscope (Photothermal Spectroscopy Corp, Santa Barbara, USA) equipped with a 40× objective (N.A. 0.78), a 4-module-pulsed QCL with a tuneable range from 1799–785 cm^−1^. The system is coupled with a Horiba Scientific iHR-320 spectrometer, 600 lines per mm grating and deep depletion CCD camera. The probe beam and Raman excitation light was from a diode laser source with 785 nm wavelength.

### Multiphoton imaging and SRS imaging

The system combines both well-established techniques SRS and SHG microscopy.^56,57^ An InSightX3 laser provides a tuneable laser between 680 and 1300 nm and a fixed laser at 1045 nm. The two beams are combined in a SF-TRU (spectral focusing – timing and recombination) unit, which allows switching between fs and ps pulses.^58^ The system is coupled to a modified confocal inverted microscope (Olympus Flouview3000 and IX81), with a 60× water objective (N.A. 1.2) and an oil condenser.

For the SRS images the tuneable beam is set to 802 nm and the delay is adjusted to excite the 2930 cm^−1^ vibrations, for TPF and SHG images the tuneable beam was set at 810 nm, with a 0.207 μm pixel size. All the data are collected using the Olympus Fluoview FV3000 software.

### Spectroscopic analysis

FTIR images of microcalcifications in samples were collected using the FTIR Agilent system. The measurements were performed in transmission mode. They are acquired between 3900–900 cm^−1^, with a spectral resolution of 4 cm^−1^ and a pixel size of 5.5 × 5.5 μm^2^. A background spectrum was recorded using a clean area of BaF_2_ for each sample. All images were created by co-adding 64 scans (for the sample) and 256 scans (for the reference). All data were collected using the Resolutions Pro software (Agilent Technologies).

A micro-Raman map of each microcalcification in the breast tissue sections was recorded using Renishaw Streamline® mode.^59^ The acquisition time was 15 seconds with a step size of 1.4 μm from 350–1800 cm^−1^ spectral range at 1500 cm^−1^ centre. Raman maps were recorded using WiRE software (Renishaw PLC).

O-PTIR and Raman single spectra were acquired simultaneously at the same locations with the same spatial resolutions (∼0.5 μm). O-PTIR spectra were collected from 1796–786 cm^−1^, with a spectral resolution of 6.6 cm^−1^ at each spectral step, using 43% of the QCL power, 59% of the probe power and 16 scans per spectrum. A background spectrum was measured using a Kevley low-E substrate prior the sample acquisition. Raman spectra were collected from a 200–2055 cm^−1^ spectral range with the grating centred at 1200 cm^−1^. Before each measurement, a system calibration was performed using three standards: silicon wafer, PTFE and aspirin.

The single IR frequency images were collected in reflective mode over with a spatial resolution of around 0.5 μm for specific frequencies of interest by tuning the QCL system at 1656, 1524, 1044 and 872 cm^−1^ for the amide I and II, phosphate and carbonate bands.

All data was collected using the PTIR Studio 4.3 software (Spectroscopy Corp., Santa Barbara, USA).

### Data analysis

All Raman spectra were pre-processed in bespoke scripts or Toolboxes in Matlab 2017 (Mathworks, USA). A cosmic ray removal was performed using a median filter, followed by a baseline correction with an asymmetric least square smoothing and a vector normalisation. K-Means cluster analysis was performed using a Toolbox in Matlab 2017 (Mathworks, USA).

### Reconstruction of SHG, TPF and SRS images

All the SRS, TPF and SHG images collected are a montage of mosaic images. A pre-processing step has been performed in order to remove the grid patterns in the images using a homemade script in Matlab 2017 (Mathworks, USA). In order to overlay the signal of both channels, a composite was created using ImageJ 1.47 software.

### Stimulated Raman histology (SRH) images

SRS images were processed in bespoke scripts^60^ or Toolboxes in Matlab 2017 (Mathworks, USA). All images were acquired using the same parameters with a field of view of 200 μm × 200 μm (512 × 512 pixels). The SRS images were acquired at 2845 and 2930 cm^−1^ for CH_2_ and CH_3_ bonds, which correspond to the lipids and proteins signal, respectively.^52,53^ The subtraction of the SRS_2930cm_^−1^ to SRS_2845cm_^−1^ images evidences the cell nuclei. By overlaying in false colour the SRS_2845cm_^−1^ and SRS_2930cm_^−1^ − SRS_2845cm_^−1^ images, it is possible to produce a virtual H&E stained image.

## Results and discussion

### Potential of the vibrational spectroscopy

**Fig. 2:**
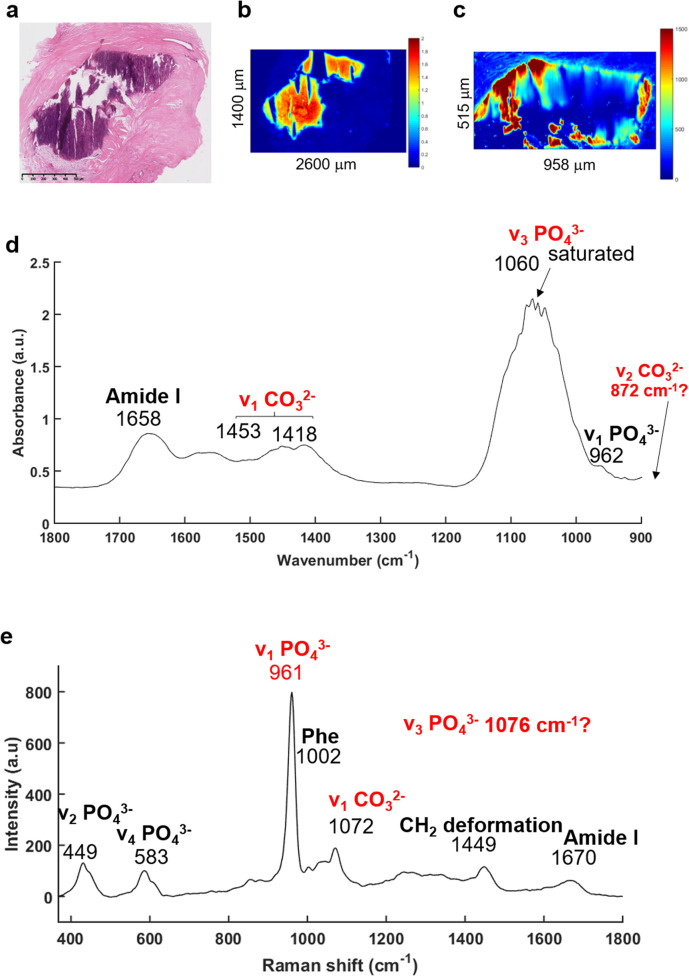
IR and Raman spectra extracted from a microcalcification. (a) White light image of the H&E stained breast tissue section with a microcalcification in the middle (purple staining) of the stroma (×5 objective), scale bar: 500 μm. (b) IR image and (c) Raman map at the phosphate peak intensity (1030 and 961 cm^−1^, respectively). (d) Mean of five IR and (e) Raman spectra were extracted within the MC, truncated onto 1800–900 cm^−1^ and 350–1800 cm^−1^, respectively.

The FTIR imaging system has been used to enable rapid screening of the BaF_2_ sections in order to localise all the BMCs, not possible using the other approaches utilised here. The IR images were then compared to the H&E section (Fig. 2). Fig. 2a illustrates a solid BMC, easy to identify, more than a millimetre in diameter.

The main advantage of the FTIR imaging system is that it consists of a large field of view (704 × 704 μm) allowing the analysis of very large images within a reasonable amount of time. In that way, it is possible to verify the presence of BMCs (based on the phosphate peak intensity) in this section, which is often very difficult to do visually as the sections are not stained. The IR image in Fig. 2b is based on the phosphate (PO_4_^2−^) peak intensity (1030 cm^−1^) showing a good correlation with the H&E section. An example of a mean IR spectrum in Fig. 2d gives information about the composition of the BMC in terms of proteins (1656 cm^−1^, amide I) and mineral content (1060 cm^−1^, phosphate band). Unfortunately, the Agilent system does not cover the long wavelength region corresponding to the carbonate (CO_3_^2−^) band at around 872 cm^−1^, when using the MCT focal plane array detector. The carbonate bands (1450–1415 cm^−1^) could be used to measure the amount of carbonate ions in the BMC but, due to possible residue of paraffin signal in this region, they should not be used alone, but in addition to those at 872 cm^−1^ for more accuracy. Furthermore, a saturation of the signal of the PO_4_^2−^ band (1060–1020 cm^−1^) is observed which is directly related to the sample thickness (as well as the inherent vibrational absorption cross-section).

In parallel, based on the IR and histological images, Raman maps have been acquired (Fig. 2c). This system has a greater spatial resolution than the FTIR system and allows selection of specific areas in the BMCs. It has been apparent for a few years that using Raman spectroscopy, the identification of minerals is relatively simple^61–64^ with the presence of the most distinct phosphate peak at 960 cm^−1^. The mean Raman spectrum illustrated in the Fig. 2e shows several phosphate bands at 961, 583 and 449 cm^−1^ corresponding to the *ν*_1_ PO_4_^2−^, *ν*_4_ PO_4_^2−^ and *ν*_2_ PO_4_^2−^ bands, respectively^65^ and are specific for mineral species. The Raman spectrum also exhibits a *ν*_1_ CO_3_^2−^ band at 1072 cm^−1^.^65^ Unfortunately, the CO_3_^2−^ peak is a low intensity shoulder in the *ν*_3_ PO_4_^2−^ band. It is difficult to determine accurately the amount of carbonate in the BMCs and validate the results obtained from the FTIR spectroscopy.

**Fig. 3:**
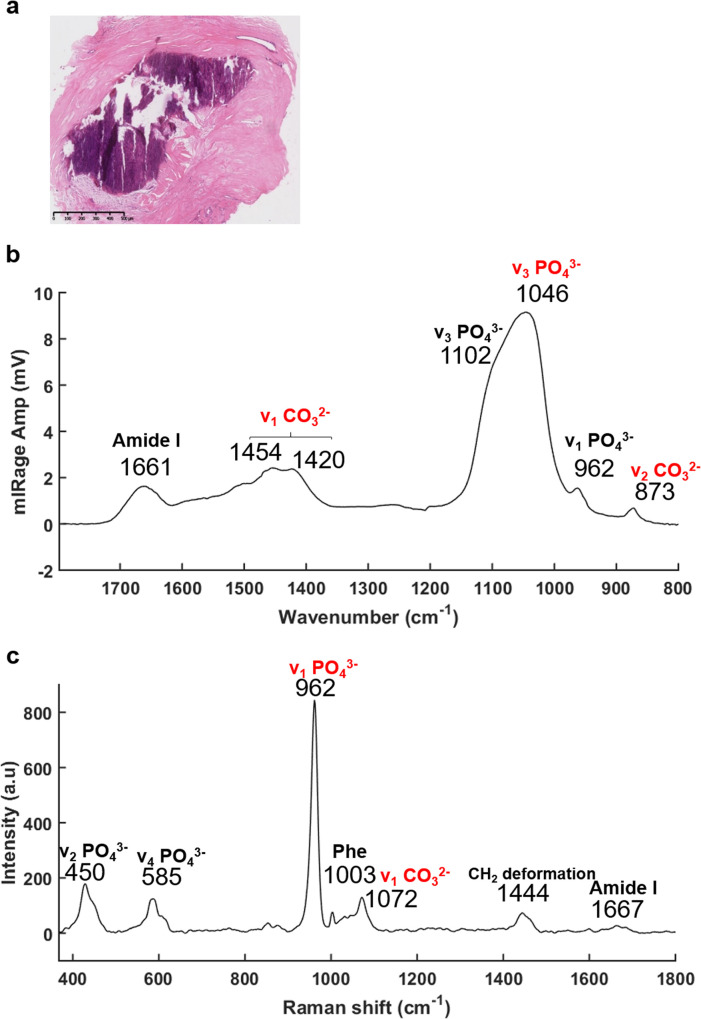
O-PTIR and Raman spectra acquired in a middle of the microcalcification, at the same time and same spot. (a) White light image of the H&E stained breast tissue section (×5 objective), scale bar: 500 μm. (b) O-PTIR and (c) Raman spectra collected within the BMC (purple staining) between 1796–786 cm^−1^ and 197–1796 cm^−1^, respectively.

The recently developed, and now commercially available, O-PTIR system (Photothermal Corp.), as outlined above, enables the simultaneous collection of IR and Raman spectra, from the same spot and same resolution.^47,66,67^ As illustrated in Fig. 3, O-PTIR and Raman spectra were collected in the centre of a solid BMC (Fig. 3a). The single O-PTIR spectrum (Fig. 3c) exhibits a phosphate peak at 1046 cm^−1^, without any saturation issues, and the carbonate band at 873 cm^−1^. In parallel, the Raman spectrum also shows phosphate and carbonate bands at 962 and 1072 cm^−1^, respectively. For the first time, we can assess and cross-validate the presence of carbonate ions in the BMCs, precisely, with both techniques.

In addition, the photothermal system, with a tuneable QCL, allows selection and acquisition of discrete wavelengths of interest with a high spatial resolution (0.5 μm per pixel). These single IR frequency images allow visualisation of the spatial distribution of proteins and lipids.

**Fig. 4:**
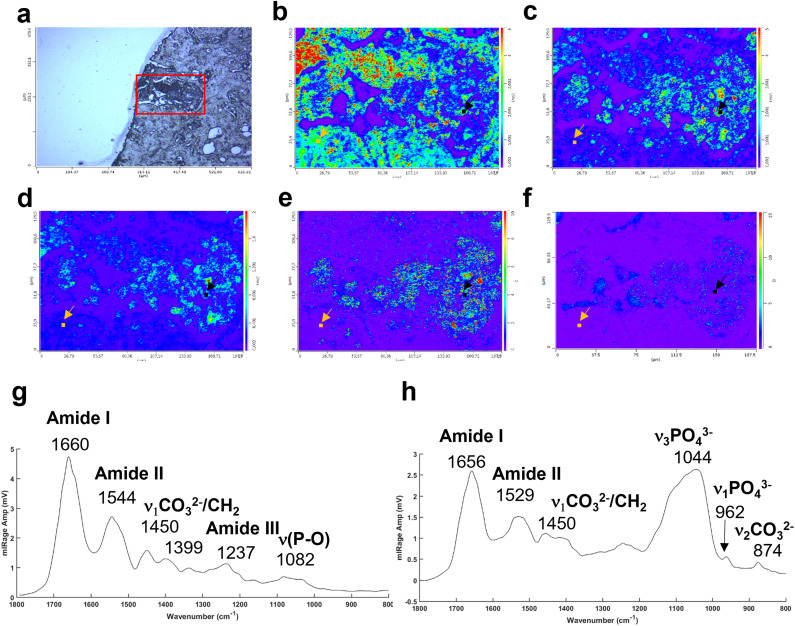
Example of a breast tissue section with BMCs analysed at different single IR wavelengths using the photothermal system. (a) White light image (×10 objective) showing a microcalcification in dark (red box). Single IR frequencies collected at (b) 1656 cm^−1^, amide I band, at (c) 1044 cm^−1^, phosphate band and (d) 872 cm^−1^, carbonate band. Illustration of different intensity ratios (e) phosphate to amide I ratio (1044 : 1656 cm^−1^) and (f) carbonate to amide I ratio (872 : 1656 cm^−1^). (g) Example of O-PTIR spectra collected from the tissue (orange dot, orange arrow) and (h) and within the BMC (black dot, black arrow).

The Fig. 4a illustrates an example of an unstained breast tissue section image containing a BMC (red box). Fig. 4b–d show spatial distribution of proteins at 1656 cm^−1^ (amide I band), 1044 cm^−1^ (phosphate band) and 873 cm^−1^ (carbonate band). The disadvantage of these discrete frequency images is that the variation of intensity across the images could reflect a difference of thickness between the BMC and the soft tissue. In order to confirm the presence of these components (*i.e.* carbonate and phosphate ions) within the BMC and the spectral features at 872 and 1044 cm^−1^, respectively, different image ratios have been performed (Fig. 4e and f) and full O-PTIR spectra were collected at specific points in the tissue and the BMC (black squares, Fig. 4g and h). The presence of the amide I, II bands (1656–1660 cm^−1^ and 1544–1529 cm^−1^) but also phosphate peak at 1044 cm^−1^ and carbonate peak 872 cm^−1^ in the O-PTIR spectra evidences the spatial distribution of these components observed in the IR single wavelength images.

### Complexity of the BMCs in breast tissue

The previous example of BMC in tissue sections illustrated in Fig. 2 was easy to identify due to its large size. Sometimes, these could be a challenge to identify when small (<100 μm) or when they are missing in the adjacent H&E section. In fact, the cutting of additional sections leads to loss of some calcifications. In others, different portions of the BMC are seen and it can be challenging to localise the BMCs. Even if the instruments have improved sensitivity or spatial resolution, when we select an area of interest, we are still limited by the white light image of unstained tissue to establish the region to image. In order to initially detect all the BMCs to enable subsequent analysis using other systems, the FTIR imaging system appears to be a good compromise for rapid screening of the tissue sections based on the phosphate peak intensity, after imaging the full spectrum at each 6.25 μm pixel. Although the O-PTIR system could be tuned to image just this peak, it would not be able to image the full tissue section (sometimes more than 2 cm length), due to its limitations in the field of view of the visible camera and the size of data that the software can process.

**Fig. 5:**
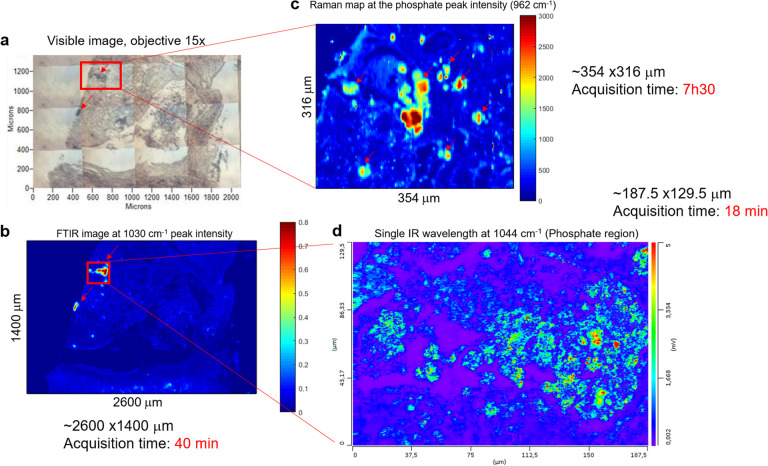
Example of breast tissue analysis combining different vibrational spectroscopic techniques. (a) White light image (×15 objective) of a BMC defining the area of the FTIR analysis. The red arrows show the BMCs. (b) Single wavelength image extracted from FTIR image of sample at the phosphate intensity at 1030 cm^−1^. The red arrows show the BMCs. (c) Raman maps based on the phosphate peak intensity at 962 cm^−1^ taken from the red square in (a). (d) O-PTIR Image at a single IR wavelength corresponding to the phosphate band (1044 cm^−1^) and taken from the red square in (b).

Fig. 5 is an example of a sample where the BMC is missing in the histological section. Based on the visible image (Fig. 5a), an IR image is recorded, highlighting two BMCs in the tissue (red arrows, Fig. 5b). Fig. 5b is a single-wavenumber image at 1030 cm^−1^ extracted from an FTIR hyperspectral data cube. A large-scale image (2.6 × 1.4 mm) is analysed in only 40 minutes. A Raman map (Fig. 5c) and an image at a single IR wavelength (1044 cm^−1^, phosphate band) (Fig. 5d) are acquired from the red square in Fig. 5a. This single frequency O-PTIR image is analysed in 20 minutes (188 × 130 μm) and illustrates a well-defined BMC using the 0.5 μm resolution. However, the acquisition time for each image depends on the size of the tissue section and the spatial resolution, which can be more important than the FTIR and Raman systems. A refinement of the area is necessary before using the photothermal system. In this process, a Raman spectroscopic analysis seems to be appropriate, by collecting a considerable image size with a satisfactory spatial resolution. In fact, the Raman map collected in Fig. 5c shows the BMC but also the soft tissue (8 hours of acquisition time for 354 × 316 μm). The Raman maps at the phosphate peak intensity (961 cm^−1^) shows several tiny BMCs surrounding the main focus of BMC in the tissue that were not visible in the IR image.

**Fig. 6:**
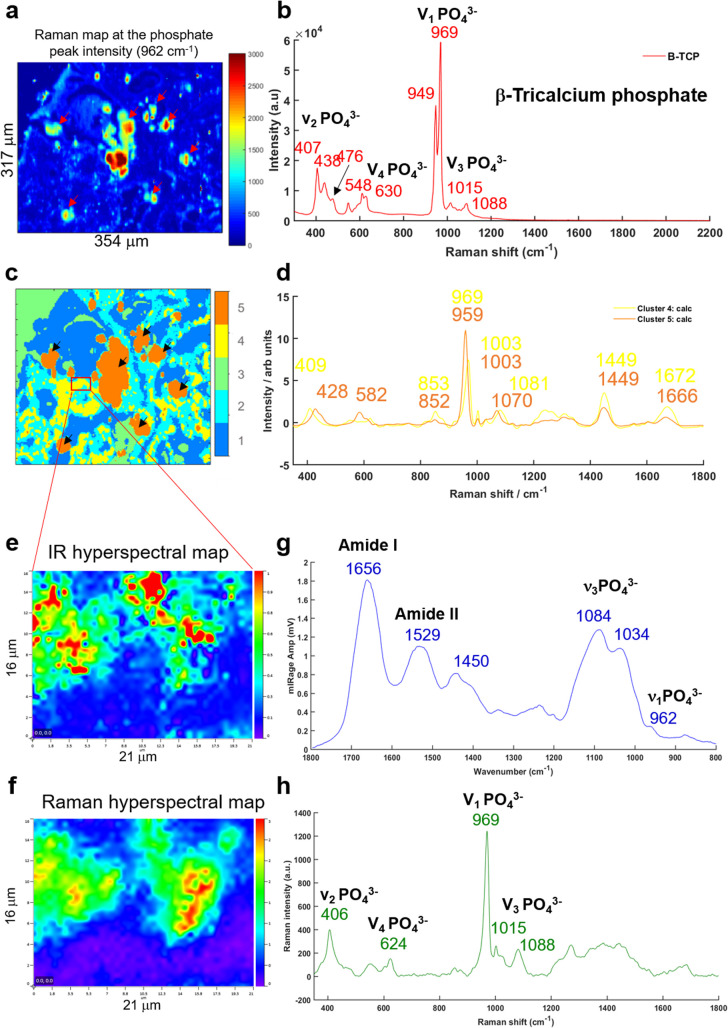
(a) Raman map of breast tissue section at the phosphate peak intensity (961 cm^−1^), the red arrows shows the BMCs in the soft tissue. (b) Single Raman spectrum of a pure mineral TCP (tricalcium phosphate) used as a reference. (c) K-Means cluster analysis with five clusters performed on the Raman map in (a). (d) Two centroids corresponding to the BMC clusters (orange and yellow in c) obtained from the K-means cluster analysis. (e) IR hyperspectral map and (f) Raman hyperspectral map from the red square in (c) at the phosphate peak intensity at 1044 and 961 cm^−1^, respectively. (g) Mean of five O-PTIR spectra and (h) mean of five Raman spectra extracted from the hyperspectral maps at the same position.

A multivariate analysis was performed on the Raman maps and illustrated in Fig. 6a and c in order to gain more information about the tissue surrounding the BMC. The K-means cluster analysis (Fig. 6c) shows different clusters exhibiting spectral features related to the tissue (dark blue and cyan clusters) and unexpectedly, two distinct clusters for the BMCs (orange and yellow clusters). In this figure, the centroids related to the tissue clusters have been removed for clarity. A comparison between the Raman map at the phosphate peak intensity and the cluster analysis evidences a good agreement. In fact, the BMCs are highlighted by the red arrows in Fig. 6a and the orange cluster in Fig. 6c. However, the multivariate analysis shows another cluster related to the BMCs represented in yellow in the Fig. 6c, diffuse in the surrounding tissue, which was not visible in the IR image in Fig. 5b. The mean spectra obtained for these two clusters illustrate different types of minerals (Fig. 6d). The orange spectrum related to the orange cluster (Fig. 6c) shows specific features for carbonated Hap with the phosphate band at 959 cm^−1^ and the carbonate band at 1070 cm^−1^, whereas the yellow spectrum exhibits two phosphate bands at 969 cm^−1^ and 1081 cm^−1^. By comparing this spectrum to our database of different standards, these BMCs are shown to be made of β-tricalcium phosphate (β-TCP). In fact, the spectrum shows the same features as the pure β-TCP component illustrated in Fig. 6b. This reflects the heterogeneity of different minerals in BMCs in the breast.

Based on the K-means image, a small area has been analysed using the Photothermal system defined in red square in Fig. 6c. Simultaneous IR and Raman hyperspectral images were recorded (16 × 21 μm for 19 hours) and illustrated in Fig. 6e and f. Regarding the acquisition time of these images, the K-means cluster analysis of the Raman maps enables targeting smaller areas of interest for the photothermal system, which are impossible to find using only the visible image, although the FTIR could also be used alone in some circumstances. The spectra extracted from the hyperspectral images at the same position show for the first time, IR and Raman spectra with characteristics of β-TCP (Fig. 6g and h). The IR spectra of different phosphate species are difficult to discriminate as they exhibit a very important *ν*_3_PO_4_^3−^ peak at around 1030 cm^−1^ combined with the organic matrix. For the first time, simultaneous IR and Raman spectra, from the same location, show specific features for the phosphate bands at 1084, 1034, 960 cm^−1^ and 969 and 1088 cm^−1^, respectively related to the β-TCP.

This study demonstrates that the combination of different vibrational spectroscopic techniques at very different spatial resolutions and imaging speeds is necessary to refine the analysis of a very specific area of interest.

### Multimodal imaging for reconstructing histological-like images

In addition to the investigation of the mineral composition of the BMCs in tissue sections, SHG and TPF microscopy and SRS spectroscopy has been performed in order to explore the structure of the collagen network associated with the microcalcifications and the proteins in the high wavenumber region (2930 cm^−1^ and 2845 cm^−1^, CH_3_ and CH_2_ bands respectively). These SRS images will reconstruction of the stimulated Raman histology (SRH) images to mimic traditional H&E staining images.

**Fig. 7:**
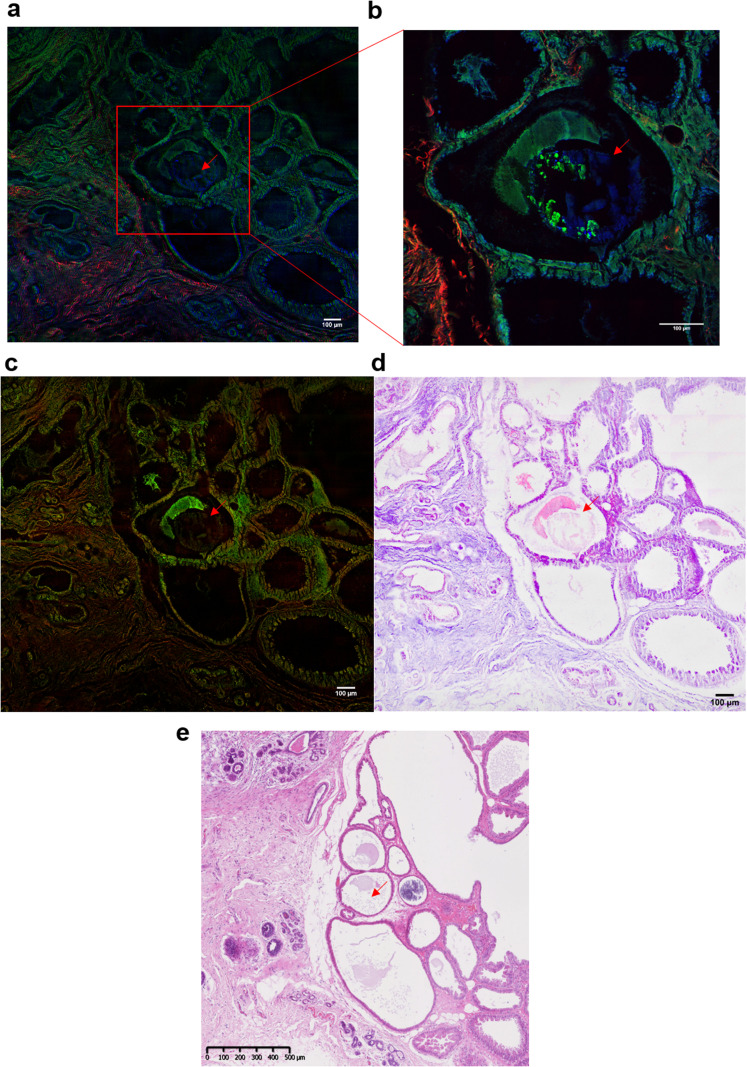
Multiphoton imaging of a breast tissue section containing a BMC. (a) Composite SRS (in green) at 2930 cm^−1^ (CH_3_ band), SHG (in red) and TPF (in blue) images of a breast tissue section containing a microcalcification (red arrow) (b) zoomed image of the region of interest delimited by the red square in (a), showing a BMC (in blue, red arrow). (c) Composite of SRS images at 2930 cm^−1^ (CH_3_ band) and 2845 cm^−1^ (CH_2_ band) (d) stimulated Raman histology image (SRH). Histology-like section reconstructed by false colour. (e) Adjacent breast tissue section was stained with H&E (×5 objective) used as reference and for comparison with the SRH.

Fig. 7 illustrates the different techniques applied to the same BMC in different tissue sections. In this example, where the BMC is missing in the H&E section (red arrow, Fig. 7e). Fig. 7a shows composite SRS (in green), TPF (in blue) and SHG (in red) images on a large field of view. This multimodal approach gives the chemical distribution of the proteins (in green) at 2930 cm^−1^ and collagen fibres in the connective tissue (in red). However, it is not possible to discriminate the different collagen types. A zoomed image of the area of interest (red square, Fig. 7a) was taken and illustrated in Fig. 7b. It demonstrates, in this tissue section that the BMC is a combination of Hap (in blue) and proteins (in green), which was confirmed by Raman spectroscopy. In order to generate the SRH image, SRS images were taken at 2930 cm^−1^ and 2485 cm^−1^ (Fig. 7c) following the protocol in different studies.^52,53^ A difference of the SRS signals (SRS_2930 cm_^−1^ − SRS_2845 cm_^−1^) was overlaid on the SRS_2845cm_^−1^ image. False colours were applied to mimic the H&E images, based on the known vibrational modes of the molecules of interest selected in the SRS. The SRS_2845cm_^−1^ image in pink representing the cytoplasm and the SRS_2930 cm_^−1^ − SRS_2845 cm_^−1^ image in purple representing the cell nuclei as illustrated in Fig. 7d. There is a good agreement between the SRH image and the traditional H&E images (Fig. 7e) even though the images are two different but adjacent tissue sections. This method allows the generation of digitally stained images by using a label-free and non-destructive technique, whilst also allowing in parallel a vibrational spectroscopic approach to gain complete information about the BMCs.

## Conclusions

This study demonstrates combined Raman and O-PTIR compositional analysis in the same location and spatial resolution on BMCs for the first time. It also shows the value of complimentary tools such as second harmonic generation to provide a high-resolution image of the collagen matrix in the BMC and adjacent tissue. The characterisation of BMCs and other pathological calcifications by O-PTIR spectroscopy has already been discussed^23,68,69^ but here, we propose a protocol for analysing BMCs using several vibrational spectroscopic and multiphoton imaging techniques. This work evidences the high potential for using the O-PTIR system for high-resolution study of the compositional distribution within BMCs. It demonstrates the presence of the carbonate band in IR and Raman (872 and 1072 cm^−1^, respectively) at the same location. Furthermore, the heterogeneity of the BMCs within the tissue is revealed, with a combination of BMCs containing Hap or β-TCP in the O-PTIR and Raman spectra. As described by Petay *et al.*, the presence β-TCP in the nanoparticles is difficult to detect using standard FTIR spectroscopy^23^ but the O-PTIR spectroscopy permitted the investigation of the presence of other mineral species, by enabling the confirmation of the results using IR and Raman spectroscopy simultaneously from the same location for the first time. This is not easy to achieve, or have confidence in, when moving the sample between different systems to measure the Raman and IR spectra. Furthermore, the O-PTIR system is not affected by the saturations seen in FTIR from the highly absorbing phosphate peaks and samples can be prepared of any thickness; so long as they are flat and fit under the microscope. In the IR, the high resolution approach also offers an advantage of visualising the spatial distribution of each sample component by tuning the QCL to select single IR frequencies of interest. Moreover, the detectors used in this system do not need to be cryogenically cooled, unlike the MCT detector required for the FTIR system.

However, there are several challenges with using the O-PTIR approach: the area of the visible image is limited by the field of view of the system (125 × 626 μm), restricting visualisation of larger tissue samples. Additionally, by increasing the spatial resolution, the acquisition time increases accordingly, which limits the ability to select large areas of interest in contrast to standard FTIR systems.

Furthermore, for some minerals, artefacts in O-PTIR spectra or a shift in the peak positions are observed *e.g.* Calcium oxalate monohydrate, cHap. This has also been shown in a study from Bazin *et al.*, suggesting that at a nanoscale analysis (AFM-IR or O-PTIR spectroscopy) there is some differences between AFM-IR and FTIR data, depending on the type of mineral analysed.^68^

Other points are noticed, for a probe at 785 nm, a sample thickness of 2, 5 or 10 μm is suitable for the O-PTIR spectroscopy analysis. However, thicker samples are recommended, in order to get an improved Raman signal. BaF_2_ or CaF_2_ slides can be used for the system, but a stainless-steel substrate is more challenging; the QCL IR source tended to heat the slide, which subsequently damaged the tissue sample. By using the stainless-steel substrate, some artefacts were observed in the O-PTIR spectra for different thicknesses of tissue sections. One hypothesis could be that this substrate reflects more than a transparent substrate, which create these artefacts. It is also possible to use glass slides, although thicker (more than 10 μm) samples are necessary to avoid the contribution of the substrate in the spectra. In the samples measured, this appears to result in BMCs more prone to burning.

The SRS and SHG techniques play an important role in the analysis for investigating the collagen content in the BMCs and further analyses are necessary to validate the results and to understand the involvement of the collagen matrix in the BMC formation. However, the presence of proteins within the calcifications tends to evidence the heterogeneous nucleation process as observed in many pathological calcifications.^70,71^ In our case, the crystal growth is embedded in the organic scaffold of proteins, DNA and collagen fibres.^70^ Moreover, this multimodal approach allows the reconstruction of SRS images to accurately mimic H&E sections. This method opens new perspectives in our scientific study to obtain faster H&E images (compare to the traditional H&E staining protocol). Furthermore, as we usually work on adjacent sections, some BMCs could be missing in the H&E sections and are hardly noticeable in unstained sections and tends to be a challenge for the vibrational spectroscopic analysis. In this case, SRS images can guide us when a BMC is missing in adjacent sections.

In conclusion, by combining a range of key complementary analytical techniques, it is possible to gain improved information regarding the spatial distribution of the mineral and organic components and the validation of the specific compositions found within BMCs.

## Author contributions

NS, KR, IL and SEP conceived the study. NS, PB and JM performed the data acquisition and data analysis. IL, SEP, EC and IB provided the breast tissue sections.

All the other authors contributed to the interpretation of the results, the writing and/or the critical revision of the manuscript and approved the final version.

## Conflicts of interest

“There are no conflicts to declare”.

## Supplementary Material
